# Interplay of ancestral non-primate lentiviruses with the virus-restricting SAMHD1 proteins of their hosts

**DOI:** 10.1074/jbc.RA118.004567

**Published:** 2018-09-04

**Authors:** Sarah A. Mereby, Tatsuya Maehigashi, Jessica M. Holler, Dong-Hyun Kim, Raymond F. Schinazi, Baek Kim

**Affiliations:** From the ‡ Department of Pediatrics, School of Medicine, Emory University, Atlanta, Georgia 30322,; § Department of Pharmacy, Kyung-Hee University, Seoul 130-701, South Korea, and; ¶ Center for Drug Discovery, Children's Healthcare of Atlanta, Atlanta, Georgia 30322

**Keywords:** lentivirus, SAM domain and HD domain-containing protein 1 (SAMHD1), macrophage, nucleotide, reverse transcription, antiviral defense, proteosomal degradation, dNTPase, immunodeficiency virus

## Abstract

Lentiviruses infect both dividing CD4^+^ T cells and nondividing myeloid cells, and the infected myeloid cells serve as long-living viral reservoirs. Host sterile alpha motif– and histidine-aspartate domain–containing protein 1 (SAMHD1) kinetically restricts reverse transcription of primate lentiviruses, including human immunodeficiency virus, type 1 (HIV-1) and simian immunodeficiency virus (SIV), in nondividing myeloid cells. SAMHD1 enforces this restriction through its dNTP triphosphohydrolase (dNTPase) activity that depletes cellular dNTPs. Some primate lentiviruses, such as HIV-2, SIVsm, and SIVagm, counteract SAMHD1 restriction by using their viral accessory proteins (Vpx or Vpr) that induce the proteosomal degradation of SAMHD1 and increase dNTP levels. SAMHD1 is conserved among non-primate mammals such as cats, cows, and horses that also carry their own lentiviruses (feline and bovine immunodeficiency viruses and equine infectious anemia viruses, respectively). However, whether these viruses also target SAMHD1 is unknown. Here, we tested whether these ancestral non-primate lentiviruses also can counteract their host SAMHD1 proteins by promoting their proteosomal degradation. Using biochemical and various cell-based assays, we observed that SAMHD1 proteins from the non-primate host species display dGTP-dependent dNTPase activity, but that the non-primate lentiviruses fail to proteosomally degrade the SAMHD1 proteins of their hosts. Our findings suggest that accessory protein–mediated proteosomal degradation of SAMHD1 did not exist among the ancestral non-primate lentiviruses and was uniquely gained by some primate lentiviruses after their transmission to primate species.

## Introduction

Lentiviruses, including human immunodeficiency virus, type 1 (HIV-1)^3^ and SIV, infect both dividing (*i.e.* activated CD4^+^ T cells) and nondividing cells (*i.e.* macrophages) during their pathogenesis (1–4), and the infected myeloid cells serve as long-living viral reservoirs that contribute to viral persistence (5, 6). However, HIV-1 rapidly replicates in activated CD4^+^ T cells, but HIV-1 replication in macrophages is kinetically suppressed (7–9). We previously reported that macrophages harbor extremely low dNTP concentrations (20–40 nm) compared with activated CD4^+^ T cells (1–5 μm) and that this low dNTP level is responsible for the restricted replication kinetics of HIV-1 in macrophages (10, 11). A series of recent studies revealed that host SAM domain– and HD domain–containing protein 1 (SAMHD1) restricts HIV-1 in myeloid cells (12, 13) by using its dNTP triphosphohydrolase (dNTPase) activity (14) and is also responsible for the low dNTP concentration found in macrophages (15, 16). Interestingly, some primate lentiviruses such as HIV-2 and SIVsm are able to replicate rapidly even in macrophages because of their accessory protein called viral protein X (Vpx) (12, 13, 17). Vpx induces the proteosomal degradation of SAMHD1 by recruiting the CUL4A/DCAF1 E3 ubiquitin ligase complex (12, 13), which elevates cellular dNTP concentration and, therefore, relieves these primate lentiviruses from suppressed replication kinetics in macrophages (15, 16). Interestingly, other SIV strains lacking Vpx, such as SIVagm, employ another viral accessory protein, Vpr, to proteosomally degrade their host SAMHD1 protein (18). However, HIV-1 Vpr cannot proteosomally degrade SAMHD1 (19), which explains why HIV-1 replication in macrophages is kinetically restricted.

Lentiviruses are also found in non-primate host species such as feline immunodeficiency virus (FIV), bovine immunodeficiency virus (BIV), and equine infectious anemia virus (EIAV). These non-primate lentiviruses also infect nondividing myeloid cells during their pathogenesis (20–23). In fact, EIAV infects exclusively macrophages in the host species (24, 25). Non-primate lentiviruses are considered as the ancestral origin of primate lentiviruses, based on the dUTPase gene encoded in non-primate lentiviruses as well as the higher diversity among FIV strains, compared with HIV-1 M strains (26). Some anti–host factor targeting functions (*i.e.* vif against APOBEC proteins) can be found in both primate and non-primate lentiviruses. However, in general, non-primate lentiviruses encode smaller numbers of accessory genes compared with primate lentiviruses.

Importantly, SAMHD1 genes are also found among mammals, including cats, cows, and horses. SAMHD1 proteins of primates (14) and mice (27, 28) were investigated for their roles in lentivirus restriction as well as for their structure and dGTP-dependent dNTPase activity. However, the dNTPase activity of SAMHD1 proteins found in the host species carrying lentiviruses has not been tested. Furthermore, it is unclear whether non-primate SAMHD1 proteins can restrict their own lentiviruses in nondividing cells and whether they are also proteosomally targeted by non-primate lentiviruses. Here, we demonstrate that although the SAMHD1 proteins of the non-primate host species harbor dGTP-activated dNTPase activity, FIV, BIV, and EIAV do not proteosomally degrade their host SAMHD1 proteins. This study demonstrates that the accessory protein–mediated SAMHD1 degradation did not exist among the ancestral non-primate lentiviruses but was established among some primate lentiviruses after lentiviruses spread to primate host species.

## Results

### dNTPase activity of feline SAMHD1 protein

**Figure 1.**
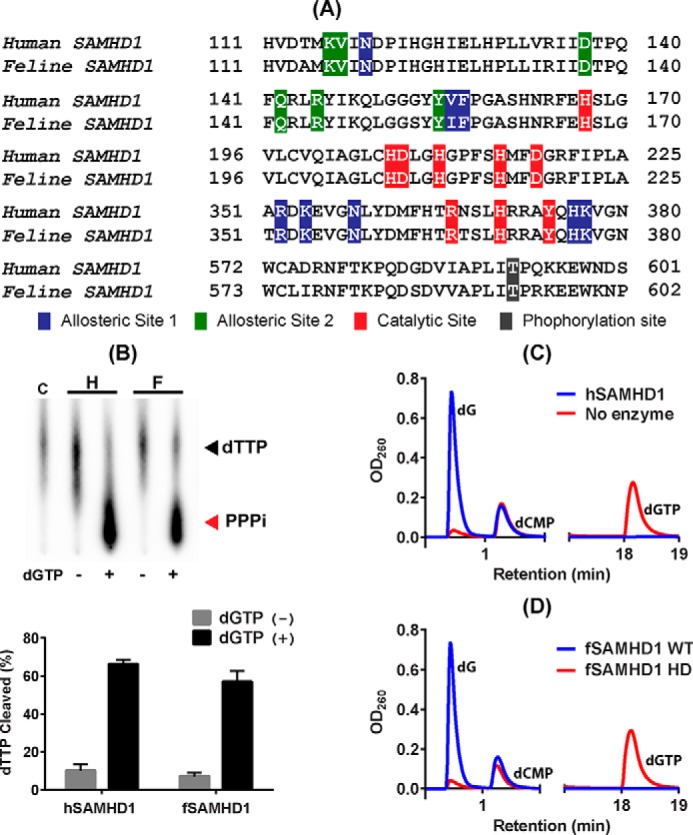
**dNTP hydrolysis activity of feline SAMHD1 protein.** *A*, sequence comparison between human and feline SAMHD1 proteins in HD active site, two allosteric sites (A1 and A2), and C-T phosphorylation site. *Red residues*, histidine/aspartate residues and residues known to be important for the dNTPase catalytic activity; *blue residues*, A1 site interacting with dGTP/GTP regulator; *green residues*, A2 site interacting with dNTPs; and *black residue*, threonine phosphorylation site (Thr-592 in hSAMHD1). *Numbers* indicate the first and last amino acid positions of the specified regions for each protein. NCBI reference sequences NM_015474.3 for hSAMHD1 and XM_003983547.2 for fSAMHD1. *B*, TLC-based assay for dGTP-dependent dTTP hydrolysis. [α-^32^P]dTTP substrate was incubated with 1 μg purified WT hSAMHD1 (*H*) and fSAMHD1 (*F*) in the presence (+) and absence (−) of dGTP for 30 min at 37 °C and heat-killed for 10 min at 70 °C, which was analyzed by TLC (32). PPPi, triphosphate product. Percentages of the cleaved dTTP substrate obtained from triplicated assays were plotted with the error bars representing S.D. *C*, HPLC-based dNTPase assay for hSAMHD1. dGTP was incubated with (*blue line*) and without (*red line*) hSAMHD1 protein for 30 min at 37 °C, and the dG product was detected after separation from dGTP substrate by HPLC. dCMP was added to each reaction as an internal loading control. *D*, HPLC-based dNTPase assay for fSAMHD1 protein. dGTP was incubated with WT fSAMHD1 (*blue line*) and fSAMHD1 HD mutant protein (*red line*). This is a representative data set from triplicates.

Although the SAMHD1 genes can be found among mammals, only primate and mouse SAMHD1 proteins were biochemically and structurally investigated for their dGTP-dependent dNTPase activity (14, 27). As shown in Fig. 1*A*, feline SAMHD1 (fSAMHD1) (sequence similarity to hSAMHD1: 77.80%) harbors the conserved histidine–aspartic acid (HD) active site for the dNTPase catalytic activity (*red*) as observed in human SAMHD1 (hSAMHD1). Both hSAMHD1 and fSAMHD1 also display strong sequence similarity at the two allosteric sites (Fig. 1*A,* A1 for *blue* and A2 for *green*) regulated by dGTP/GTP and dNTPs, respectively (29). Additionally, the C-terminal Thr-592 phosphorylation site (*black* in Fig. 1*A*), which is important for the cell cycle–mediated regulation of its dNTPase activity (30, 31), is also conserved between hSAMHD1 and fSAMHD1 proteins.

First, we biochemically tested whether fSAMHD1 protein harbors the dGTP-activated dNTPase activity. GST-tagged fSAMHD1 HD domain was overexpressed in *Escherichia coli* and purified by using GST column chromatography followed by GST-tag removal and gel filtration. We also constructed and purified the catalytically inactive HD dNTPase active site mutant of fSAMHD1 (H206R D207N). Initially, both of the purified WT and HD mutant fSAMHD1 proteins, which showed high purity (Fig. S1), were tested for their dGTP-dependent dNTPase activity by using TLC-based assay (32). In this assay, [α-^32^P]dTTP (substrate) was incubated with SAMHD1 proteins in the presence or absence of an activator, dGTP. As shown in Fig. 1*B*, the ^32^P-triphosphate product (PPPi) of the radioactive dTTP substrate hydrolysis by hSAMHD1 (*H*) and fSAMHD1 (*F*) was detected only when incubated with dGTP, and more than 60% of the initial dTTP substrate was hydrolyzed under the reaction condition. These data suggest that fSAMHD1 requires dGTP for dTTP hydrolysis as observed with hSAMHD1. We additionally confirmed the dNTPase activity of fSAMHD1 by using HPLC-based assay (32). In this assay, dGTP works both as an activator for the A1 and A2 sites and as a substrate of SAMHD1, and, dG, which is a product of dGTP hydrolysis, was separated from dGTP by HPLC. As shown in Fig. 1*C*, when dGTP was incubated with (*blue line*) or without (*red line*) hSAMHD1, the elevated amount of the dG product was detected only for the incubation with hSAMHD1. dCMP was used as an internal loading control in this assay. When dGTP was incubated with the purified WT (*blue line*) or HD mutant (*red line*) fSAMHD1 proteins (Fig. 1*D*), only WT fSAMHD1 produced the elevated dG product levels. Overall, the data in Fig. 1 demonstrate that fSAMHD1 is a dGTP-dependent dNTPase as observed with hSAMHD1.

### Effect of fSAMHD1 expression on cellular dNTP levels in nondividing cells

**Figure 2.**
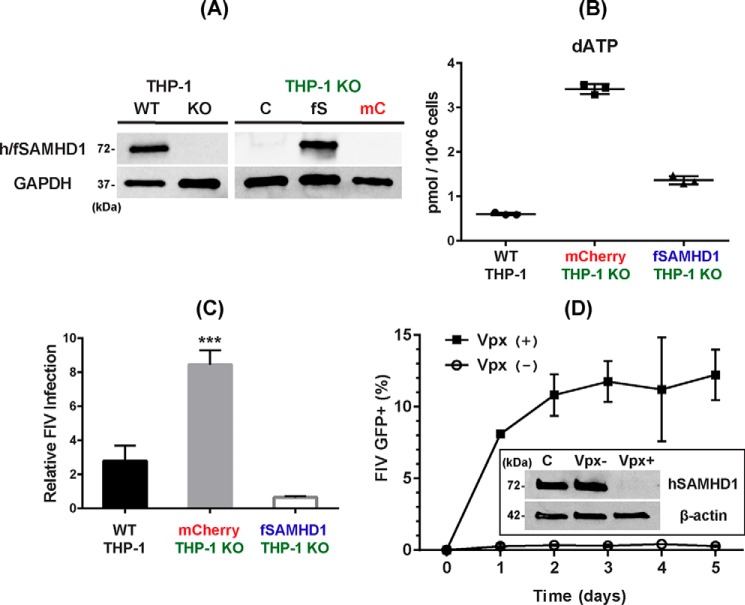
**Cellular dNTP reduction activity and antiviral activity of fSAMHD1 in nondividing THP-1 macrophages.** *A*, expression of fSAMHD1 in hSAMHD1 KO THP-1 cells. *Left*, human SAMHD1 expression in the PMA-treated parental THP-1 macrophages (WT) was previously abolished by CRISPR/Cas9 (KO) (33). hSAMHD1 expression was detected by Western blotting with anti-SAMHD1 antibody in the differentiated THP-1 cells. Cellular GAPDH protein was used as a loading control. *Right*, hSAMHD1 KO THP-1 cells (THP-1 KO) were transduced with lentiviral vector co-expressing both HA-tagged fSAMHD1 (*fS*) and mCherry protein or lentiviral vector expressing only mCherry protein (*mC*), and the mCherry+ cells were FACS-sorted. The sorted mCherry+ THP-1 were then differentiated to macrophages by PMA for 7 days and analyzed for the fSAMHD1 expression (*fS*) by Western blotting with HA antibody. GAPDH was used as a loading control. C, untransduced SAMHD1 KO THP-1 control cells. *B*, cellular dNTP reduction by fSAMHD1 expression in nondividing hSAMHD1 KO THP-1 macrophages. The parental THP-1 cells expressing hSAMHD1 (WT THP-1), hSAMHD1 KO THP-1 cells expressing only mCherry protein (mCherry THP-1 KO), and hSAMHD1 KO THP-1 cells expressing both fSAMHD1 and mCherry protein (fSAMHD1 THP-1 KO) were differentiated by PMA for 7 days, and the dNTP levels in these cells were determined by the RT-based dNTP assay. The total dNTP amounts were normalized by 1 × 10^6^ cells. dATP levels are shown in this figure, and other three dNTP data are in Fig. S2. *C*, FIV restriction by fSAMHD1 in nondividing hSAMHD1 KO THP-1 macrophages. The parental THP-1 cells expressing hSAMHD1 (WT THP-1), hSAMHD1 KO THP-1 cells expressing only mCherry protein (mCherry THP-1 KO), and hSAMHD1 KO THP-1 cells expressing HA-tagged fSAMHD1 were differentiated by PMA, and transduced with an equal amount of FIV-GFP vector with *error bars* representing S.D. ***, *p* ≤ 0.001, using two-tailed Student's *t* test. The -fold changes of the GFP+ cells compared with the untransduced THP-1 negative background control cells (ratio = 1) in triplicates were plotted. The transduction efficiencies of the vectors used (% GFP ± S.D.) were 1.187 ± 0.384, 3.567 ± 0.361, and 0.813 ± 0.101 for WT THP-1, mCherry THP-1 KO, and fSAMHD1 THP-1 KO, respectively. *D*, FIV restriction by hSAMHD1 in human primary monocyte-derived macrophages. Human primary monocyte-derived macrophages prepared from four healthy donors were pretreated with VLPs with (Vpx+) or without Vpx (Vpx−) for 12 h, and then transduced with an equal amount of FIV-GFP vector. The transduced cells were collected every day for 5 days, and the percent of the GFP+ cells in triplicates was determined by FACS. Insert shows Western blotting conducted to confirm the degradation of hSAMHD1 by Vpx in human primary macrophages: β-actin was used as a loading control, and antiSAMHD1 antibody was used for detecting hSAMHD1 in the human primary macrophages. C, no VLP pretreatment control macrophages.

Next, we tested whether the expression of fSAMHD1 in nondividing cells reduces cellular dNTP concentrations. For this test, we employed SAMHD1 knockout (KO) human monocytic THP-1 cells that we previously established by CRISPR/Cas9 (33). Fig. 2*A* shows the loss of hSAMHD1 expression in the PMA-mediated differentiated SAMHD1 KO THP-1 cells, compared with the parental WT THP-1 cells. First, we transduced the SAMHD1 KO THP-1 cells with a lentiviral vector co-expressing both HA-tagged fSAMHD1 and mCherry protein. The mCherry-positive cells, which also express fSAMHD1, were then FACS sorted and propagated. Next, we tested the expression of the HA-tagged fSAMHD1 in the propagated mCherry-positive SAMHD1 KO THP-1 cells, after PMA-mediated differentiation (7 days) to nondividing macrophages, by Western blotting. As shown in Fig. 2*A*, the mCherry-positive SAMHD1 KO THP-1 cells (*fS*) showed fSAMHD1 expression, whereas the same cells transduced with the control vector expressing only mCherry protein (*mC*) as well as untransduced control KO cells (*C*) showed no fSAMHD1 expression.

Next, we measured the dNTP levels in the differentiated SAMHD1 KO THP-1 cells with or without fSAMHD1 expression. We previously reported that SAMHD1 KO THP-1 cells showed increased cellular dNTP levels, compared with the non-KO parental WT THP-1 cells, after the PMA-mediated differentiation (33). As shown in Fig. 2*B*, the differentiated SAMHD1 KO THP-1 cells expressing only mCherry showed higher dATP levels, compared with the differentiated parental WT THP-1 cells. However, this elevated dATP level was reduced by the expression of fSAMHD1 (dATP in Fig. 2*B* and other three dNTPs in Fig. S2), supporting that fSAMHD1 works as dNTPase in the nondividing cells as predicted by its biochemical dNTPase activity (Fig. 1*B*).

### Anti-FIV activity of fSAMHD1 in nondividing cells

Next, we evaluated the antiviral activity of fSAMHD1 in differentiated/nondividing SAMHD1 KO THP-1 cells. For this test, we compared the transduction efficiency of the FP93-based and VSV-G–pseudotyped FIV vector expressing GFP (34) in the PMA-differentiated SAMHD1 KO THP-1 cells with or without fSAMHD1 expression by FACS analysis. This FIV vector system employs pFP93 packaging plasmid encoding FIV *gag*, *pol*, and *rev* genes, but not *vif* and *tat*-like *orfA,* as well as *env*. As shown in Fig. 2*C*, the SAMHD1 KO THP-1 cells showed the increased FIV-GFP vector transduction efficiency, compared with the parental WT THP-1 cells, which is because of the elevated dNTP concentration induced by loss of hSAMHD1 as we reported previously (33). However, as shown in Fig. 2*C*, the differentiated SAMHD1 KO THP-1 cells that express fSAMHD1 displayed reduced transduction efficiency of the FIV-GFP vector, compared with the same SAMHD1 KO cells without fSAMHD1 expression (mCherry protein only).

Next, we also tested whether human SAMHD1 can restrict FIV vector in human primary macrophages. For this test, human primary monocyte-derived macrophages from four healthy donors were pretreated with virus-like particles (VLPs) with (+) or without (−) Vpx that degrades hSAMHD1. Indeed, as shown in the insert of Fig. 2*D*, we confirmed the complete degradation of hSAMHD1 in human primary monocyte-derived macrophages by Vpx. Next, human primary macrophages were transduced with FIV-GFP vector, and the transduction efficiency was determined by FACS analysis for GFP expression. As shown in Fig. 2*D*, human macrophages treated with Vpx (+) VLPs greatly enhanced the transduction efficiency of FIV-GFP vector, compared with the macrophages treated with Vpx (−) VLPs. This suggests SIV Vpx can render primary human macrophages permissive to FIV replication, most likely because of Vpx-mediated degradation of hSAMHD1. Overall, the data presented in Fig. 2 demonstrate that fSAMHD1 reduces cellular dNTP concentration and restricts FIV in nondividing THP-1 cells as hSAMHD1 restricts HIV-1 in macrophages.

### Test for proteosomal fSAMHD1 degradation by FIV

**Figure 3.**
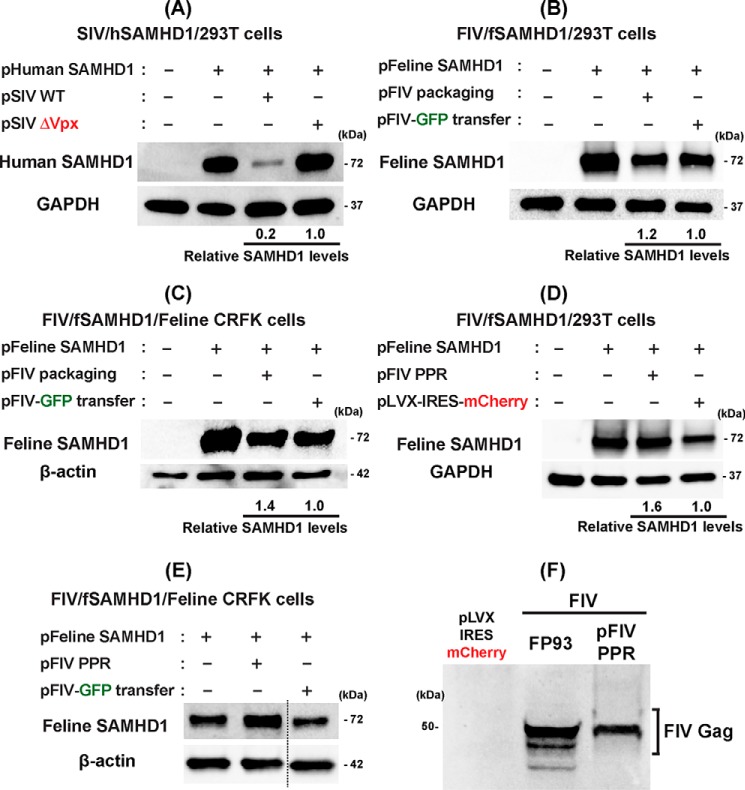
**Test for fSAMHD1 degradation by FIV.** *A*, degradation of hSAMHD1 by SIV. Human 293T cells were co-transfected with pLVX-IRES-mCherry co-expressing HA-tagged hSAMHD1 and mCherry protein (*pHuman SAMHD1*) (0.1 μg) and a plasmid expressing SIVmac251 proteins either with (*pSIV WT*) (2 μg) or without Vpx (*pSIV* Δ*Vpx*) (2 μg), and the hSAMHD1 levels were determined by Western blotting with anti-hSAMHD1 antibody. GAPDH was used as a loading control. Transfection efficiency for the cells expressing hSAMHD1 were monitored by the mCherry protein expression. *B*, test for the degradation of fSAMHD1 by FIV. 293T cells were co-transfected with a plasmid co-expressing HA-tagged fSAMHD1 and mCherry (*pFeline SAMHD1*) (0.1 μg) and either FIV packaging plasmid expressing FIV protein except Env protein, FP93 (*pFeline packaging*) (2 μg) (34) or FIV transfer plasmid expressing only GFP, pGINSIN FIV-GFP (*pFIV-GFP transfer*) (2 μg). fSAMHD1 levels were visualized by HA antibody. Transfection efficiency was monitored by the mCherry expression. *C*, test for the degradation of fSAMHD1 by FIV in feline CRFK cells. Feline CRFK cells were co-transfected with the fSAMHD1 expressing plasmid and either FP93 packaging plasmid or pGINSIN FIV-GFP transfer plasmid as described in *B*. The fSAMHD1 expression was visualized by HA antibody, and cellular β-actin was used as a loading control. *D*, test for the degradation of fSAMHD1 by infectious molecular clone of pFIV-PPR. The experiment described in (*B*) was repeated except using full-length FIV-PPR molecular clone. pLVX-IRES-mCherry was used as a negative control. *E*, the experiment described in (*C*) was repeated except using full-length FIV-PPR molecular clone. The fSAMHD1 expression was visualized by HA antibody, and cellular β-actin was used as a loading control. The data presented in this figure are representative data from more than three independent transfections, and mean relative SAMHD1 levels shown were calculated by densitometry analysis. The calculated mean ± S.D. values are (*A*) 0.21 ± 0.05, (*B*) 1.21 ± 0.49, (*C*) 1.40 ± 0.57, and (*D*) 1.60 ± 0.2. *F*, detection of FIV Gag protein in the 293T cells transfected with FIV plasmids. The same lysates prepared from 293T cells transfected with FP93 and pFIV-PPR used in (*B*) pFIV packaging and in (*D*) pFIV PPR were used to detect FIV Gag protein expression with anti-FIV gag antibody. Cells transfected with pLVX-IRES-mCherry expressing only mCherry protein were used as a negative control.

We then tested whether FIV is capable of proteosomally degrading fSAMHD1, as HIV-2 and some SIV strains degrade hSAMHD1. For this test, we employed the SAMHD1 degradation assay established previously (18, 35). In this assay, cells are co-transfected with plasmids expressing (i) SAMHD1 protein and (ii) viral proteins, and then the SAMHD1 protein levels were assessed by Western blotting. First, to validate the SAMHD1 degradation assay, we examined the degradation of hSAMHD1 by SIVmac251 with or without Vpx, which was reported previously (12, 13). Human kidney epithelial 293T cells were co-transfected with a pLVX-IRES-mCherry plasmid (0.1 μg) co-expressing HA-tagged hSAMHD1 and mCherry protein and pSIV3 expressing SIVmac251 proteins either with Vpx (2 μg, pSIV WT) or without Vpx (2 μg, pSIV ΔVpx) (36, 37). The SAMHD1 protein levels in these transfected cells were determined by Western blotting with anti-HA antibody (or anti-hSAMHD1 antibody for hSAMHD1 level), and cellular GAPDH protein was used as a loading control. An equal transfection efficiency among transfections was monitored by mCherry expression from the SAMHD1 expression plasmid (data not shown). As shown in Fig. 3*A*, the SIVmac251 protein expression including Vpx (pSIV WT) reduced hSAMHD1 protein levels in 293T cells whereas the SIVmac251 protein expression without Vpx failed to reduce hSAMHD1. The SAMHD1 level reduction is because of the proteosomal degradation mediated by Vpx as reported previously (13).

Next, we tested the fSAMHD1 degradation by FIV by using the same SAMHD1 degradation assay. For this test, we co-transfected 293T cells with pLVX-IRES-mCherry plasmid expressing HA-tagged fSAMHD1 along with mCherry protein and either (i) FIV vector packaging plasmid, FP93 FIV (pFIV packaging) (34), which expresses FIV proteins except the Env protein, or (ii) FIV-GFP transfer plasmid, pGINSIN FIV-GFP (pFIV-GFP transfer) (34), which expresses only GFP. As shown in Fig. 3*B*, no significant decrease of the fSAMHD1 protein was detected in the cells co-transfected with pFIV packaging plasmid, compared with the cells co-transfected with the pFIV-GFP transfer plasmid, suggesting that FIV does not proteosomally degrade fSAMHD1 in 293T cells.

Because it is possible that the failure of the fSAMHD1 degradation by FIV in 293T cells is because of the potential host specificity difference of the CLT4/DCAF4 E3 ligase system, which is essential to the SAMHD1 degradation, we next repeated the assay for the fSAMHD1 degradation by FIV in feline kidney epithelial cell line, CRFK (instead of human kidney epithelial 293T cells). Again, as shown in Fig. 3*C*, the FIV packaging plasmid was not able to degrade fSAMHD1 in this feline cell line, suggesting that the failure of the fSAMHD1 degradation by FIV is not because of the host specificity of the ubiquitin-mediated protein degradation.

We repeated the fSAMHD1 degradation assay by employing a full-length molecular clone of FIV PPR. Indeed, as shown in Fig. 3*D*, we also did not observe the fSAMHD1 degradation by the full-length FIV PPR clone in the transfected 293T cells. In addition, the full-length molecular clone of FIV PPR failed to reduce the fSAMHD1 level in CRFK cells (Fig. 3*E*). Importantly, we confirmed the expression of FIV proteins in the 293T cells transfected with the FIV protein expressing plasmids (FP93 FIV packaging plasmid and pFIV PPR) by detecting the FIV Gag protein expressed from these FIV plasmids (Fig. 3*F*), suggesting that the failure of the FIV-mediated fSAMHD1 degradation is not because of the failure of the FIV protein expression in 293T cells. Collectively, the data shown in Fig. 3 support that FIV does not proteosomally degrade its feline host SAMHD1 protein.

### Test for proteosomal degradation of bSAMHD1 by BIV and eSAMHD1 by EIAV

**Figure 4.**
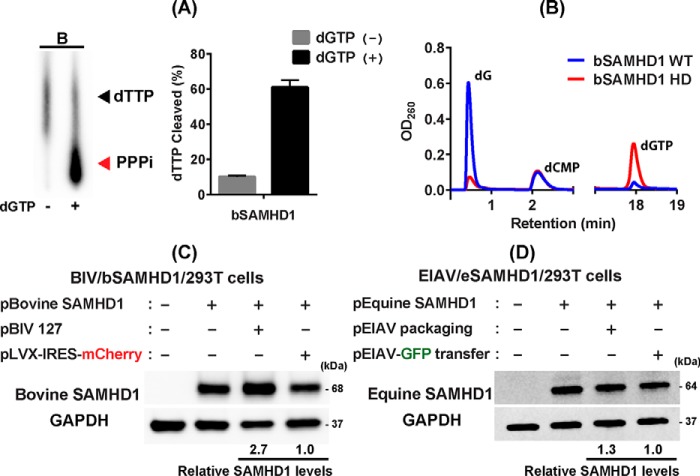
**Test for bSAMHD1 degradation by BIV and eSAMHD1 degradation by EIAV.** *A* and *B*, TLC-based (*A*) and HPLC-based (*B*) dNTPase activity assays of bSAMHD1 protein. Both TLC- and HPLC-based dNTPase assay were conducted with purified bSAMHD1 protein. These assays were performed in triplicates as described for fSAMHD1 in Fig. 1. [α-^32^P]dTTP and dGTP were used as SAMHD1 substrates in TLC- and HPLC-based assays, respectively. PPPi, triphosphate product. The HPLC data show the dGTP hydrolysis and dG product by WT bSAMHD1 (*blue line*) and HD mutant (*red line*). dCMP is the internal loading control. *C*, test for degradation of bSAMHD1 by BIV. Human 293T cells were co-transfected with pLVX-IRES-mCherry co-expressing HA-tagged bSAMHD1 and mCherry protein (*pBovine SAMHD1*) (0.1 μg) and either a full-length molecular clone of BIV (*pBIV 127*) (2 μg), or pLVX-IRES-mCherry expressing on mCherry protein (*pLVX-IRES-mCherry*) (2 μg). The bSAMHD1 protein levels were determined in Western blotting with HA antibody. GAPDH was used as a loading control. *D*, test for degradation of eSAMHD1 by EIAV. Human 293T cells were co-transfected with pLVX-IRES-mCherry co-expressing HA-tagged eSAMHD1 and mCherry protein (*pEquine SAMHD1*) (0.1 μg) and either EIAV vector packaging plasmid (*pEIAV packaging*) (2 μg) or GFP-expressing EIAV vector transfer plasmid (*pEIAV-GFP transfer*) (2 μg). The eSAMHD1 protein levels were determined by Western blotting with HA antibody. GAPDH was used as a loading control. The Western blotting data presented in this figure are representative data from more than three independent transfections, and mean relative SAMHD1 levels shown were calculated by densitometry analysis. The calculated mean ± S.D. values are (*C*) 2.69 ± 0.40 and (*D*) 1.29 ± 0.27.

Next, we investigated whether two other non-primate lentiviruses, BIV and EIAV, proteosomally degrade their own host SAMHD1 proteins (bSAMHD1 and eSAMHD1, respectively). First, we tested the dNTPase activity of bSAMHD1 protein which also shows conserved HD active site residues as well as two A1 and A2 regulatory sites (Fig. S3), compared with hSAMHD1 (sequence similarity to hSAMHD1: 76.83%). We overexpressed and purified bSAMHD1 protein in *E. coli* with high purity (Fig. S1*B*) and tested its biochemical dGTP-dependent dNTPase activity by TLC- and HPLC-based assays as described in Fig. 1 for fSAMHD1. As shown in Fig. 4*A*, the purified bSAMHD1 protein was able to hydrolyze dTTP in a dGTP-dependent manner in TLC-based assay as assessed by the PPPi production only in the presence of dGTP. WT bSAMHD1 protein (Fig. 4*B*, *blue line*) was also able to hydrolyze dGTP as confirmed by the elevated level of the dG product in the HPLC-based assay, whereas the catalytically inactive HD active site bSAMHD1 mutant (H194R D195N) (Fig. 4*B*, *red line*) protein failed to hydrolyze dGTP substrate.

Then we investigated the proteosomal degradation of bSAMHD1 by BIV. For this test, we co-transfected 293T cells with pLVX-IRES-mCherry plasmid co-expressing HA-tagged bSAMHD1 and mCherry protein and either a full-length infectious clone of BIV, pBIV127, or pLVX-IRES mCherry control. As shown in Fig. 4*C*, the transfection with pBIV127 did not reduce bSAMHD1 protein level, suggesting that as observed with HIV-1 and FIV, BIV also does not proteosomally target its host SAMHD1 protein.

We next tested whether EIAV can proteosomally degrade eSAMHD1. Although it is highly likely that eSAMHD1 is also dNTPase from the sequence comparison (Fig. S3) (sequence similarity to hSAMHD1: 82.34%), the dNTPase activity of eSAMHD1 protein could not be tested biochemically because unlike other SAMHD1 proteins tested in this study, eSAMHD1 was insoluble during the protein expression and purification. Finally, to test the eSAMHD1 degradation by EIAV, we co-transfected 293T cells with pLVX-IRES-mCherry plasmid co-expressing HA-tagged eSAMHD1 and mCherry protein and either EIAV vector packaging plasmid expressing EAIV proteins (pEIAV packaging, pEV53D (38)) or GFP expressing EIAV vector transfer plasmid (pEIAV-GFP transfer, pEIAV-SIN6.1 CGFPW (39)). As shown in Fig. 4*D*, the expression of EIAV protein from the EIAV packaging plasmid did not reduce the eSAMHD1 level, compared with pEIAV-GFP control. Collectively, the data shown in Fig. 4 demonstrate that as observed with FIV, both BIV and EIAV also do not proteosomally target their own host SAMHD1 proteins.

### Test for the degradation of non-primate host SAMHD1 proteins by Vpx

**Figure 5.**
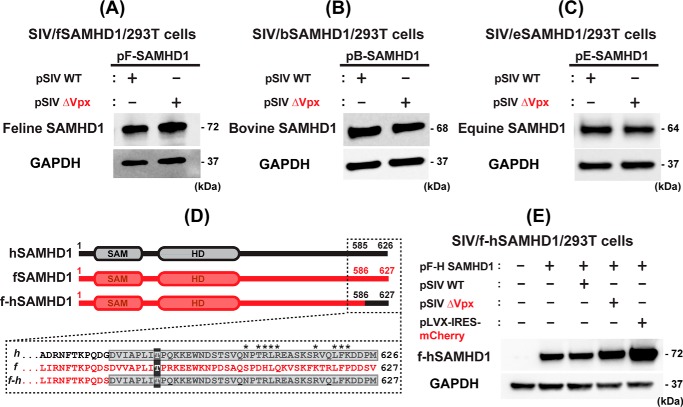
**Test for non–primate host SAMHD1 protein degradation by Vpx and fSAMHD1 C-T region for Vpx recognition.** *A–C*, tests for Vpx-induced degradation of the non-primate host SAMHD1 proteins. 293T cells were transfected with pLVX-IRES-mCherry co-expressing HA-tagged SAMHD1 proteins from the three non-primate host species (*A*, fSAMHD1; *B*, bSAMHD1; and *C*, eSAMHD1) (0.1 μg) and either pSIV WT (2 μg) or pSIV ΔVpx (2 μg), and the SAMHD1 protein levels were determined by Western blotting with HA antibody. GAPDH was used as a loading control. *D*, construct of fSAMHD1 chimeric construct (*f-hSAMHD1*) containing the C-terminal Vpx recognition region of hSAMHD1. The sequence of the hSAMHD1 C-terminal region recognized by Vpx as well as the sequence of fSAMHD1 at the equivalent region is in *dotted box. Asterisk* sites are the positions of hSAMHD1 depicted for the Vpx recognition (PDB ID: 4CC9). The *numbers* indicate the amino acid positions of each SAMHD1. The f-hSAMHD1 chimera was constructed by swapping the fSAMHD1 C terminus (residues 586–627) with hSAMHD1 C terminus (residues 585–626) containing Vpx recognition region. The C-term phosphorylation sites (Thr-592 and Thr-593 in human and feline SAMHD1, respectively) are shaded in *dark gray* for reference. *E*, test for the degradation of f-hSAMHD1 chimeric construct by SIV. 293T cells were co-transfected with pLVX-IRES-mCherry co-expressing HA-tagged f-hSAMHD1 hybrid construct and mCherry protein (*pF-H SAMHD1*) (0.1 μg) and either pSIV WT (2 μg) or pSIV ΔVpx (2 μg). pLVX-IRES-mCherry expressing only mCherry protein (2 μg) was used as a negative control. The f-hSAMHD1 chimeric protein was detected by HA antibody. The Western blotting data presented in this figure are representative data from more than three independent transfections.

Finally, we tested whether Vpx can proteosomally degrade the SAMHD1 proteins from the three non-primate host species. As shown in Fig. 5*A–C*, when 293T cells were co-transfected with the non–primate host SAMHD1-expressing plasmids with either pSIV WT or pSIV ΔVpx, both cells transfected with pSIV WT and pSIV ΔVpx displayed the same levels of the host SAMHD1 proteins (Fig. 5*A*, fSAMHD1; Fig. 5*B*, bSAMHD1; and Fig. 5*C*, eSAMHD1), suggesting that Vpx does not degrade these non–primate host SAMHD1 proteins. The expression and function of SIV Vpx in our experimental system was validated by the reversal of Vpx-mediated hSAMHD1 degradation when treated with proteasome inhibitor MG132 (Fig. S4).

Vpx primarily recognizes the C-terminal region of hSAMHD1 (Fig. 5*D*) even though this SAMHD1 recognition also needs the N-terminal sequences of primate SAMHD1 proteins in some cases (35, 40). The positions marked with an *asterisk* in Fig. 5*D* are the Vpx interaction sites depicted from the SIV Vpx-SAMHD1 X-ray crystal structure available (PDB ID: 4CC9) (41). The failure of the fSAMHD1 degradation by Vpx (Fig. 5*A*) could be explained by the limited conservation at those sites known to be important for the Vpx recognition in hSAMHD1 (see the sequence at Fig. 5*D*). Therefore, we investigated whether introducing Vpx recognition sequence from hSAMHD1 into fSAMHD1 can induce the fSAMHD1 degradation by Vpx. For this test, we constructed a fSAMHD1 chimeric protein by swapping the fSAMHD1 C-terminal end (residue 586–627) with hSAMHD1 C terminus (residue 585–626) containing the Vpx recognition sequence (f-hSAMHD1) (Fig. 5*D*). For this test, when 293T cells were transfected with pLVX-IRES-mCherry expressing the HA-tagged f-hSAMHD1 chimeric protein and either pSIV WT and pSIV ΔVpx, the levels of the f-hSAMHD1 chimeric protein remained the same regardless of Vpx (Fig. 5*E*), suggesting that regions other than the known C-terminal Vpx recognition sequence are also required to induce the fSAMHD1 protein degradation by Vpx. Collectively, these data support the host-specific SAMHD1 recognition of Vpx, as observed previously among SAMHD1 proteins of primate host species (35).

## Discussion

Non-primate lentiviruses have been considered as ancestral origins of primate lentiviruses: the dUTPase gene encoded among non-primate lentiviruses is not found in primate lentiviruses, and the loss of the dUTPase gene is a key genetic clue for claiming non-primate lentiviruses as the origin of primate lentiviruses (26, 42, 43). Additionally, larger phylogenic distances between non-primate lentiviruses, compared with the distances between primate lentiviruses, support that primate lentiviruses originated from a non-primate lentivirus (26). Possibly, a single transmission of a non-primate lentivirus to primate species initiated the spreading of the primate lentiviruses (26).

Initially, SAMHD1 was identified as a nondividing myeloid-specific host restriction factor against primate lentiviruses including HIV-1 and SIV strains. The dNTP depletion capability of SAMHD1 with its dNTPase activity is a key antiviral mechanism against lentiviruses. We and others demonstrated that SAMHD1 also displays antiviral activity against several dsDNA viruses (44) and hepatitis type B virus (45) possibly through its dNTPase activity. Some primate lentiviruses, however, are able to counteract host SAMHD1 by using their accessory proteins such as Vpx (*i.e.* HIV-2 and SIVsm) or Vpr (SIVagm strains), which allows these primate lentiviruses to replicate rapidly in nondividing myeloid cells. However, Vpr encoded in HIV-1 strains cannot induce the proteosomal degradation of hSAMHD1 (19). This explains their suppressed replication kinetics in nondividing myeloid cells, compared with that in activated CD4^+^ T cells and of SIV strains that can proteosomally degrade SAMHD1 in macrophages. The cellular dNTP level impact on the replication kinetics of non-primate lentiviruses in dividing *versus* nondividing cells has not been investigated. Also, there is no genome analysis report that the non-primate lentiviruses studied here encode Vpx-like accessory proteins. Therefore, considering the inability to proteosomally degrade their host SAMHD1 protein (Figs. 3 and 4) and the capability of fSAMHD1 to suppress FIV replication in nondividing cells (Fig. 2*C*), it is plausible that non-primate lentiviruses replicate slowly in nondividing cells containing low dNTP concentrations, compared with dividing cells with higher dNTP concentrations, as observed with HIV-1 strains.

Our data validated the SAMHD1 genes found in cats and cows encode the expected dGTP-dependent dNTPase proteins. All three SAMHD1 proteins from the non-primate host species tested here display highly conserved sequences in the HD dNTPase active site as well as A1 and A2 allosteric regulatory sites (Fig. S3) (sequence similarity to hSAMHD1: fSAMHD1, 77.80%; bSAMHD1, 76.83%; eSAMHD1, 82.34%; calculated by Clustal 2.1) (46). However, some interesting variations among these SAMHD1 proteins were observed. First, the C-terminal end of SAMHD1 encodes a conserved threonine residue (Thr-592 in human SAMHD1) that plays a key role in the negative regulation of its dNTPase activity through phosphorylation, and cyclin-dependent kinases were known to phosphorylate this threonine residue, resulting in the inactivation of the dNTPase activity, particularly in dividing cells (30). However, bSAMHD1 C-terminal sequence does not encode the conserved threonine residue (see *black* sequence in Fig. S3). It is possible that bSAMHD1 employs an alternative residue for its phosphorylation-mediated regulation of its dNTPase activity. However, whether these ancestral lentiviruses counteract their own host SAMHD1 proteins by phosphorylation or any unknown alternative mechanisms, instead of proteosomal degradation, remains to be tested. Second, bSAMHD1 has a slightly shorter SAM domain, compared with the other SAMHD1 protein tested in this study. Finally, eSAMHD1 has a 68 amino acid deletion between the HD active site and the A1 regulatory site, compared with other SAMHD1 proteins, which may have contributed to its difference in solubility as observed during purification in this study.

Overall, our data support that the accessory protein–mediated and proteosomal degradation–based anti-SAMHD1 mechanism did not exist among the ancestral non-primate lentiviruses and suggest that some primate lentiviruses (*i.e.* SIVsm and SIVagm) gained the anti-SAMHD1 mechanism engineered by their accessory proteins (Vpx or Vpr) after lentiviruses spread to primate host species, whereas HIV-1 failed to establish the accessory protein–mediated anti-SAMHD1 strategy.

## Experimental procedures

### Plasmids, cells, lentiviral vectors, and SAMHD1 genes

pGEX-5X-1 (GE Healthcare) and pLVX-IRES-mCherry (Clontech) were used for the expression of SAMHD1 proteins in *E. coli* and mammalian cells, respectively. The following full-length clones of BIV and FIV were obtained through the AIDS Reagent Program, Division of AIDS, NIAID, National Institutes of Health: pBIV127 from Drs. Charles Wood and M. A. Gonda and FIV PPR infectious molecular clone (pFIV-PPR) from Dr. John Elder. FIV packaging plasmid, FP93 FIV, and transfer plasmid, pGINSIN FIV-GFP, were kindly provided by Dr. Eric Poeschla (University of Colorado, Denver, CO) (34). pSIV3, plasmid expressing SIVmac251 except Env, as well as pSIV3 with Vpx deletion, pSIV ΔVpx, (36, 37) were obtained from Dr. Nathaniel Landau (New York University, New York, NY). EIAV packaging plasmid, pEV53D (38), and GFP-expressing EIAV transfer plasmid, pEIAV-SIN6.1 CGFPW (39), were obtained from Dr. J. C. Olsen (Addgene plasmid numbers 44168 and 44171, respectively). 293T cells (Invitrogen), human monocytic THP-1 cells, and CRFK (ATCC) as well as human primary monocyte-derived macrophages were used in this study. Human monocyte-derived macrophages were prepared from monocytes isolated from buffy coats of four healthy donors as described previously (11). SAMHD1 KO THP-1 cells were previously constructed by CRISPR/Cas9 (33). SAMHD1 KO THP-1 cells expressing fSAMHD1 were constructed by transduction with pLVX-IRES-mCherry–based lentiviral vector co-expressing HA-tagged fSAMHD1 and mCherry protein, followed by FACS sorting for mCherry-positive cells for propagation. Lentiviral vectors used in this study were prepared by co-transfecting packaging and transfer plasmids as well as plasmid expressing VSV-G envelope protein to 293T cells as described previously (11). SIVmac251 based VLPs with and without Vpx were prepared from 293T cells as described previously (47). The SAMHD1 sequences described in this study were obtained from NCBI reference sequences: NM_015474.3 (human SAMHD1), NM_001075861.1 (bovine SAMHD1), XM_003983547.2 (feline SAMHD1), and XM_008541885.1 (equine SAMHD1). The hSAMHD1 gene encoded from the plasmid provided by Dr. Felipe Diaz-Griffero (30) was cloned into pLVX-IRES-mCherry with the N-terminal end HA and FLAG tandem tag. The genes of feline, bovine, and equine SAMHD1s (fSAMHD1, bSAMHD1, and eSAMHD1, respectively) were synthesized in pGEX-5X-1 by GenScript (Piscataway, NJ) for *E. coli* expression and then cloned to pLVX-IRES-mCherry (XhoI and NotI sites) for mammalian expression with N-terminal HA tag.

### Expression and purification of SAMHD1 proteins

The N-terminal GST-fused full-length human, feline, bovine, and equine SAMHD1 proteins were overexpressed in Rosetta DE3 cells (Novagen) by inducing with 0.2 mm IPTG at optical density (600 nm) of 0.5–0.8 for 48 h at 16 °C. Cells were harvested by centrifugation at 4000 × *g* for 30 min, followed by sonication in lysis buffer containing 40 mm Tris HCl, pH 7.5, 250 mm KCl, 5% glycerol, 0.1% Triton X-100, 5 mm β-mercaptoethanol (β-Me), 0.1 mm PMSF, and 0.5 mm benzamidine. Cleared lysate was obtained by centrifugation at 39,000 × *g* and applied to a GSTrap FF column (GE Healthcare) that had been equilibrated with binding buffer containing 50 mm Tris-HCl, pH 7.5, 10% glycerol, 250 mm KCl, and 5 mm β-Me. The column was washed for 20 column volume (CV) with the binding buffer, followed by 5 CV wash with increased KCl (1–2 m final). The column was re-equilibrated for 5 CV with protease cleavage buffer containing 50 mm Tris-HCl, pH 8.0, 20% glycerol, 250 mm KCl, and 5 mm β-Me and 2 mm CaCl_2_, and Factor Xα (New England Biolabs) was added (50 units/CV) to the column and allowed to cleave 24–48 h on-column at 4 °C. The protein was then eluted with the protease cleavage buffer. Fractions containing GST tag–free SAMHD1 were combined and further purified on Superdex S200 10/300 (GE Healthcare) with gel-filtration buffer containing 50 mm Tris HCl, pH 7.5, 20% glycerol, 150 mm KCl, 1 mm β-Me, and 0.25 mm EDTA, and the fraction containing SAMHD1 was combined and flash frozen in liquid nitrogen and stored in −80 °C until use. eSAMHD1 protein was found to be insoluble during the purification. The purified GST tag–free full-length fSAMHD1, bSAMHD1 proteins and hSAMHD1 protein are >95% pure judged by SDS-PAGE (Fig. S1) and used for the dNTPase assay.

### Thin layer chromatography–based dNTPase assay

A mixture of 0.01 μCi/μl [α-^32^P]dTTP and 200 μm unlabeled dTTP was incubated with or without 200 μm dGTP and 1 μg SAMHD1 proteins in the SAMHD1 reaction buffer containing 50 mm Tris-HCl, pH 7.5, 50 mm KCl, 5 mm MgCl_2_ for 60 min at 37 °C. Reaction was heat-inactivated by incubating for 10 min at 65 °C. The radioactive dTTP substrate and radioactive triphosphate product were separated by TLC by spotting the reaction mixture (0.7 μl) on a PolyGram 300 CEL TLC plate (Macherey-Nagel) with the mobile phase containing 0.8 m LiCl and 50 mm Tris-HCl, pH 7.5, and quantitated as described previously (32).

### HPLC-based SAMHD1 dNTPase assay

Reactions contained 1 μm purified SAMHD1 protein and 1 mm dGTP in the SAMHD1 reaction buffer. Reactions were initiated with the addition of 1 μg SAMHD1, incubated for 1 h at 37 °C, and terminated by incubation for 10 min at 65 °C. Reactions were diluted in 12.5% acetonitrile containing 1 mm dCMP as a loading control and then injected into a Beckman Coulter System Gold 126 solvent module as described previously (32). dG product abundance was monitored for the dGTPase activity. No enzyme control and HD SAMHD1 mutant control were used for comparison of the dG product amounts.

### Establishment of SAMHD1 KO THP-1 cells expressing fSAMHD1

We previously reported THP-1 cells with and without human SAMHD1 expression, which were established by CRISPR/Cas9 (33). These cells were transduced with pLVX-IRES-mCherry–based vector expressing only mCherry protein or both mCherry and HA-tagged fSAMHD1, and the mCherry+ cells were FACS-sorted and expanded. The fSAMHD1 expression in these cells was detected after PMA-mediated differentiation by Western blotting with anti-HA antibody (Abcam, Cambridge, MA).

### Cellular dNTP measurement

dNTP levels in THP-1 cells were measured by HIV-1 RT-based dNTP assay (11). Briefly, cellular dNTPs were extracted from an equal number of cells (typically 2 × 10^6^ cells) by 60% methanol. The dried dNTP samples were re-suspended in water for the reverse transcriptase–based primer extension reaction, which determines the amounts of dNTPs in the extracted samples. The dNTP amounts were normalized by 1 × 10^6^ cells.

### SAMHD1 degradation assay

The SAMHD1 degradation assay was conducted as reported previously (18, 35). Briefly, 293T cells or CRFK cells (1 × 10^6^ cells) were co-transfected with both plasmid-expressing (2 μg) viral proteins or control proteins (mCherry protein or GFP) and pLVX-IRES-mCherry plasmids (0.1 μg) co-expressing HA-tagged SAMHD1 proteins and mCherry protein by polyethylenimine (PEI). The cell lysates were prepared by sonication from the transfected cells at 48 h post transfection and applied to Western blots to visualize HA-tagged SAMHD1 proteins by anti-HA antibody. β-actin or GAPDH was used for the loading control. We also monitored FIV Gag protein in the transfected cells by using anti-FIV Gag antibody (from Dr. Eric Poeschla, University of Colorado) to confirm the FIV protein expression. For testing the reversal of Vpx-induced degradation of hSAMHD1 by proteasome inhibitor MG132 (Fig. S4), MG132 was added 2 h prior to the transfection (1 μm final) and kept at the same concentration throughout the experiment. Cells were harvested 48 h post transfection and the hSAMHD1 levels were determined by Western blotting with anti-hSAMHD1 antibody. Transfection efficiency for the cells expressing hSAMHD1 were monitored by the mCherry protein expression.

## Author contributions

S. A. M., T. M., and J. M. H. data curation; S. A. M., T. M., J. M. H., and B. K. formal analysis; S. A. M., T. M., and J. M. H. validation; S. A. M., T. M., and J. M. H. visualization; S. A. M. and T. M. methodology; S. A. M., T. M., J. M. H., and B. K. writing-review and editing; D.-H. K., R. F. S., and B. K. conceptualization; D.-H. K., R. F. S., and B. K. resources; R. F. S. and B. K. funding acquisition; B. K. supervision; B. K. investigation; B. K. writing-original draft.

## Supplementary Material

Supporting Information

## Footnotes

3 The abbreviations used are:

HIV-1: human immunodeficiency virus, type 1
SIV: simian immunodeficiency virus
dNTPase: dNTP triphosphohydrolase
FIV: feline immunodeficiency virus
BIV: bovine immunodeficiency virus
EIAV: equine infectious anemia virus
PPPi: inorganic triphosphate
PMA: phorbol 12-myristate 13-acetate
VLP: virus-like particle
β-Me: β-mercaptoethanol
CV: column volume.
