# Supplementary material for: Interplay of ancestral non-primate lentiviruses with the virus-restricting SAMHD1 proteins of their hosts
Source: J Biol Chem. 2018 Sep 4;293(42):16402–12. doi: 10.1074/jbc.RA118.004567 (PMC6200947; doi:10.1074/jbc.RA118.004567)
Supplement: Supporting Information [file supp_293_42_16402__index.html]

Interplay of ancestral non-primate lentiviruses with the virus-restricting SAMHD1 proteins of their hosts — Non-primate lentiviruses and host SAMHD1 proteins — Supporting Information 

# Interplay of ancestral non-primate lentiviruses with the virus-restricting SAMHD1 proteins of their hosts

## Supporting Information

- Supporting Information (to be published online) - Supplementary figures
