# Supplementary material for: Interplay of ancestral non-primate lentiviruses with the virus-restricting SAMHD1 proteins of their hosts
Source: J Biol Chem. 2018 Sep 4;293(42):16402–12. doi: 10.1074/jbc.RA118.004567 (PMC6200947; doi:10.1074/jbc.RA118.004567)
Supplement: Supporting Information [file supp_RA118.004567_138966_2_supp_194711_pcbrcc.pdf]

Figure S1

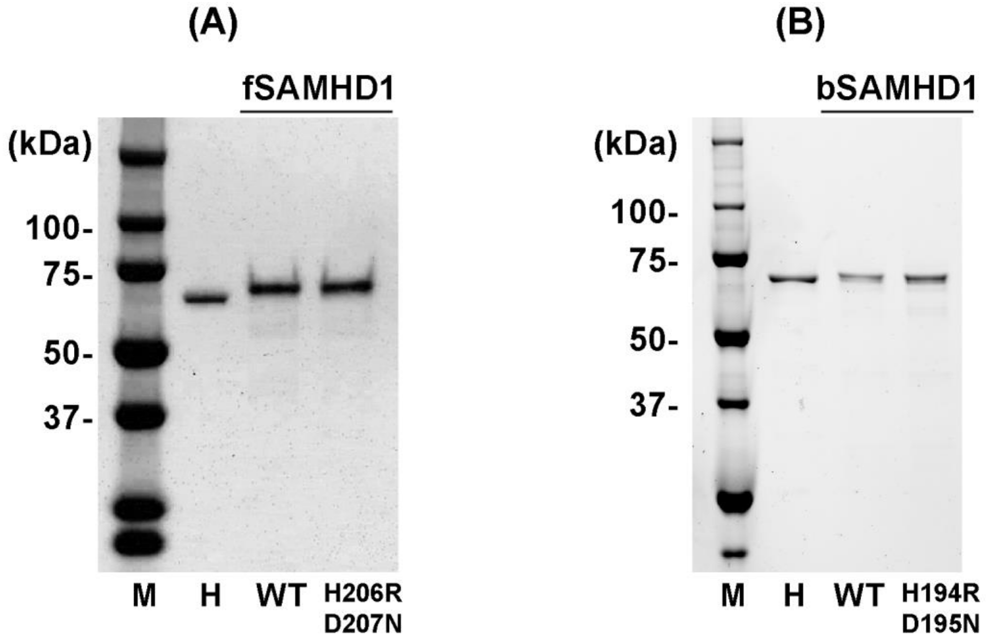

Figure S1: Purified SAMHD1 proteins in *E. coli*. The purified GST tag free full-length fSAMHD1 **(A)** and bSAMHD1 **(B)** proteins were analyzed in SDS-PAGE. M: Protein molecular weight markers, H: purified hSAMHD1 (tag free, full-length), WT: wild type SAMHD1 proteins, and HD: catalytically inactive active site mutant SAMHD1 proteins.

Figure S2

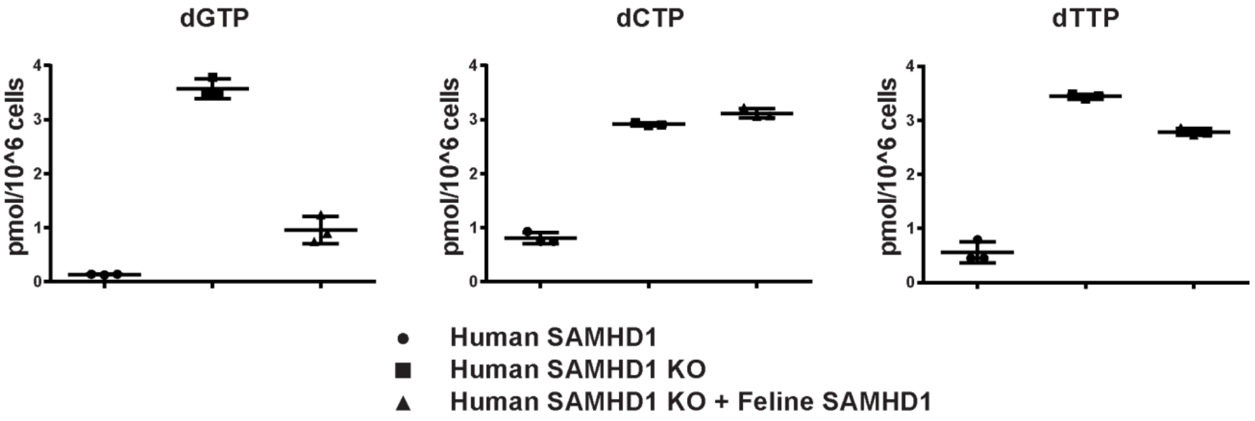

Figure S2: dNTP level in THP-1 cell expressing fSAMHD1. The levels of dGTP, dCTP and dTTP in the differentiated parental THP-1 cells with human SAMHD1 (circle), differentiated human SAMHD1 KO THP-1 cells (square), and differentiated human SAMHD1 KO THP-1 cells expressing feline SAMHD1 expression (triangle) were determined by the RT-based dNTP assay in triplicates as described for dATP measurement (Figure 2B). The error bars; SDs.

Figure S3

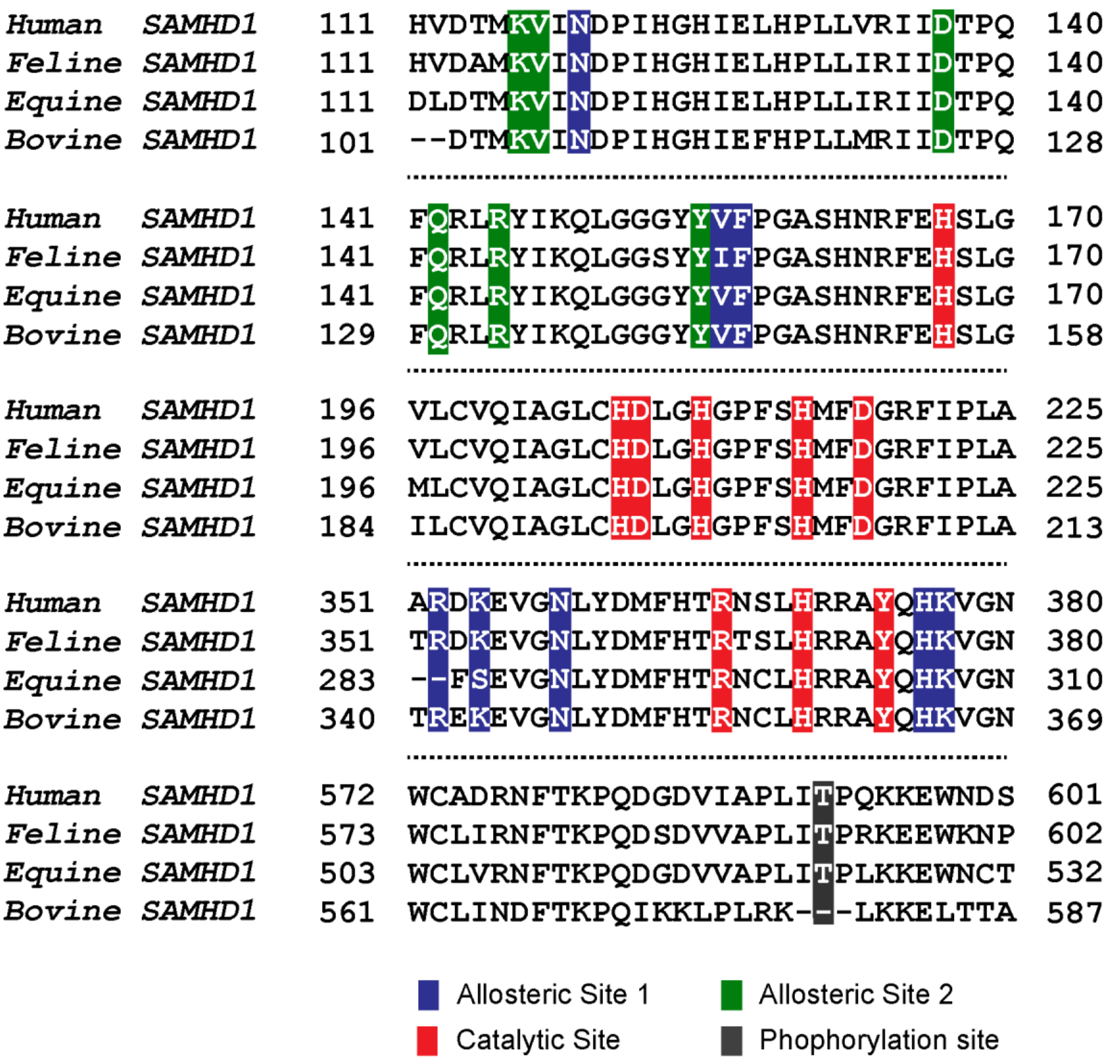

Figure S3: Sequence comparison of human, feline, bovine and equine SAMHD1 proteins. The amino acid sequences of the HD active site (red), A1 (blue) and A2 (green) allosteric sites, and C-terminal phosphorylation sites (dark grey) of the four SAMHD1 proteins were compared. The sequence sources are: NCBI reference sequences: NM_015474.3 (hSAMHD1), XM_003983547.2 (fSAMHD1), NM_001075861.1 (bSAMHD1), and XM_008541885.1 (eSAMHD1).

Figure S4

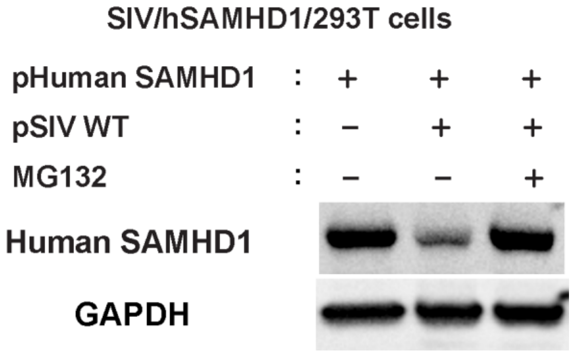

Figure S4: Reversal of the Vpx induced degradation of hSAMHD1 by MG132. Human 293T cells were co-transfected with pLVX-IRES-mCherry co-expressing HA-tagged hSAMHD1 and mCherry protein (“pHuman SAMHD1”, 0.1µg) and a plasmid expressing SIVmac251 proteins with (“pSIV WT”, 2µg). In order to verify functional Vpx expression, MG132 was added (1µM, final) 2hrs prior to the transfection and kept at the same concentration throughout the experiment. Cells were harvested 48 hours post transfection, and the hSAMHD1 levels were determined by western blots with anti-hSAMHD1 antibody. GAPDH was used as a loading control. Transfection efficiency for the cells expressing hSAMHD1 were monitored by the mCherry protein expression.
